# Expression Analysis of Heavy-Chain-Only Antibodies in Cloudy Catshark and Japanese Bullhead Shark

**DOI:** 10.3390/md23010028

**Published:** 2025-01-08

**Authors:** Reo Uemura, Susumu Tanimura, Nao Yamaguchi, Ryuichi Kuroiwa, Gabriel Takashi Andrés Tsutsumi, Toshiaki Fujikawa, Kiyoshi Soyano, Kohsuke Takeda, Yoshimasa Tanaka

**Affiliations:** 1 Department of Cell Regulation, Graduate School of Biomedical Sciences, Nagasaki University, Bunkyo-machi 1-14, Nagasaki 852-8521, Japantakeda-k@nagasaki-u.ac.jp (K.T.); 2 Institute for East China Sea Research, Organization for Marine Science and Technology, Nagasaki University, 1551-7, Taira-machi, Nagasaki 851-2213, Japan; f-toshiaki@nagasaki-u.ac.jp (T.F.); soyano@nagasaki-u.ac.jp (K.S.); 3 Center for Medical Innovation, Nagasaki University, Sakamoto 1-7-1, Nagasaki 852-8588, Japan

**Keywords:** cloudy catshark, heavy-chain-only antibody, immunoglobulin new antigen receptor, Japanese bullhead shark, VNAR-domain antibody

## Abstract

Heavy chain-only antibodies in sharks are called immunoglobulin new antigen receptors (IgNAR), consisting of one variable region (VNAR) and five constant regions (C1-C5). The variable region of IgNAR can be expressed as a monomer composed of a single domain, which has antigen specificity and is thus gaining attention as a next-generation antibody drug modality. In this study, we analyzed IgNAR of the cloudy catshark and Japanese bullhead shark, small demersal sharks available in the coastal waters of Japan. By analyzing the IgNAR gene sequence and comparing it with the constant regions of five other known shark species, high homology was observed in the C4 region. Consequently, we expressed the recombinant protein of the C4 domain from the cloudy catshark in *E. coli*, immunized rats, and produced antibodies. The obtained antiserum and mAbs recognized the C4 recombinant protein of the cloudy catshark, but reacted minimally with the plasma of non-immunized cloudy catsharks and instead reacted with the plasma of Japanese bullhead sharks. The results of this study imply that the protein expression levels of IgNAR in cloudy catsharks may be relatively lower compared to those in Japanese bullhead sharks, however, this interpretation remains to be determined through further studies.

## 1. Introduction

Modern drug discovery has been ongoing for about 130 years since the late 19th century. Initially, the targets for drug discovery were receptors that recognize small molecule agonists expressed on cell membranes and intracellular enzymes that catalyze small molecules as substrates. As a result, many drugs were small molecule antagonists or inhibitors. The development of these small molecule drugs was greatly facilitated by the establishment of large compound libraries and high-throughput screening systems. However, since the human genome consists of only about 20,000 genes, the number of these drug discovery targets is limited to at most a few thousand. In fact, by the late 20th century, approximately 100 years after modern drug discovery began, the depletion of drug discovery targets had become a problem.

Meanwhile, from the end of the 20th century, attention began to focus on cell membrane receptors that recognize protein ligands as new drug targets. As a result, extracellular protein–protein interactions have attracted attention as drug targets. Protein–protein interactions typically have large interaction surfaces, making it difficult for conventional small molecule drugs to sufficiently inhibit these interactions. Furthermore, there are often no pockets on the interaction sites for small molecules to bind to. Therefore, attention has shifted from small molecules to antibodies, which are larger, more specific, and have broader interaction surfaces, as a modality for drug discovery.

In fact, from the late 20th century to the early 21st century, antibody drugs have dominated the main pipelines of drug discovery and development as biopharmaceuticals [1,2]. However, since these protein receptors and ligands involved in extracellular protein–protein interactions are also encoded by genes, they are just a part of the approximately 20,000 genes, amounting to at most a few thousand. Consequently, to create new drugs, it is necessary to move to new drug targets. Among these, intracellular protein–protein interactions are gaining attention as new drug targets. While normal antibodies are sufficient to block extracellular protein interactions, they cannot penetrate the cell membrane to inhibit intracellular protein interactions. Thus, there is a need to develop antibodies smaller than the approximately 150 kDa antibody molecules [3].

As part of these efforts, single-chain antibodies, where the variable regions derived from the heavy and light chains of antibody molecules are linked by a linker, are being developed [4,5]. For single-chain antibodies to exhibit antigen specificity, the variable regions of the heavy and light chains must bind together, requiring hydrophobic amino acid side chains at the binding site. Generally, when hydrophobic regions are exposed on the molecular surface, physicochemical stability decreases, leading to reduced stability of single-chain antibodies. Therefore, single-domain antibodies, which are expressed as recombinant proteins from the variable regions of heavy-chain-only antibodies found in animals such as camels and cartilaginous fish, are currently receiving much attention [6,7]. Nanobodies derived from camelids are smaller than single-chain antibodies and exhibit a greater variability in the length of complementarity-determining region 3. This enables them to target protein sites through alternative interaction mechanisms, due to their distinct topology, which conventional antibodies cannot access. Additionally, nanobodies offer enhanced physicochemical stability, while also reducing both manufacturing complexity and associated costs. These attributes position nanobodies as superior to conventional antibody therapeutics.

In camelids such as dromedary camels, alpacas, and llamas, heavy-chain-only antibodies IgG2 and IgG3 exist [8,9,10]. These heavy-chain only antibodies consist of a variable domain (VHH) and constant domains CH2 and CH3. Since VHH consists of a single domain and can recognize antigens as a monomer, recombinant proteins of VHH are being developed as nanobodies [11,12,13,14,15].

Cartilaginous fish such as sharks and rays also have heavy-chain-only antibodies IgNAR (immunoglobulin new antigen receptor), and their variable region VNAR (variable domain of shark new antigen receptor) can be expressed as monomers similar to VHH from camelids [16,17,18]. Additionally, our university hosts both the Department of Fishery and the Institute for East China Sea Research, which are equipped with well-established facilities for fish husbandry and aquaculture. Given the relatively straightforward rearing and cultivation of small shark species, we chose to use sharks as immunological model animals. Sharks possess IgM and IgW antibodies composed of heavy and light chains that are antigen-responsive, and IgNAR also shows increased antigen-specific antibody titers upon antigen sensitization [19,20,21,22]. Recent years have seen active research into applying these for antibody drug development [6,7,23,24,25,26,27].

Sharks are classified into 9 orders with over 500 species [28]. To date, the analysis of shark antibodies has primarily focused on relatively large shark species, such as the nurse shark (*Ginglymostoma cirratum*) with a body length of 200–300 cm. Studies involving smaller shark species have also been conducted [23,27,29]. In our research group, we selected the cloudy catshark (*Scyliorhinus torazame*, ~50 cm in body length) and the Japanese bullhead shark (*Heterodontus japonicus*, 50–120 cm in body length) as experimental animals. These species are relatively easy to obtain in the coastal waters of Japan and are docile and easy to handle. While the genomic configuration of immunoglobulin genes in mammals is translocon configuration, in sharks, it is cluster configuration, and the number of clusters varies among shark species [18,21,22,30,31]. Therefore, the expression level of IgNAR may vary by shark species, and it is expected that the response of IgNAR to antigen sensitization will also differ significantly among species.

To develop shark-derived single-domain antibodies utilizing shark VNARs, it is crucial to find sharks that show a marked IgNAR response to antigen sensitization or determine the conditions for such antigen sensitization. In this study, monoclonal antibodies against IgNAR were established, and the expression of IgNAR was analyzed using the cloudy catshark (*Scyliorhinus torazame*) and Japanese bullhead shark (*Heterodontus japonicus*), small demersal (benthopelagic or bottom-dwelling) sharks inhabiting near the bottom of the coastal waters of Japan.

## 2. Results

### 2.1. Genetic Sequence Analysis of Heavy-Chain-Only Antibodies Derived from Cloudy Catshark

We initially searched for cloudy catshark gene sequences (*Scyliorhinus torazame*, Family: *Scyliorhinidae*, Order: *Carcharhiniformes*, Storazame v1.0) in the database of RIKEN BDR (Squalomix: elasmobranch sequence archive by Laboratory for Phyloinformatics (Kuraku Lab)), which show high homology with the heavy-chain-only antibody (IgNAR) gene sequences of nurse shark (*Ginglymostoma cirratum*, Family: *Ginglymostomatidae*, Order: *Orectolobiformes*, GenBank: U18701.1), spotted wobbegong (*Orectolobus maculatus*, Family: *Orectolobidae*, Order: *Orectolobiformes*, GenBank: DQ268538.1), banded houndshark (*Triakis scyllium*, Family: *Triakidae*, Order: *Carcharhiniformes*, GenBank: AB557743.1), small-spotted catshark (*Scyliorhinus canicular*, Family: *Scyliorhinidae*, Order: *Carcharhiniformes*, GenBank: JX556022.1), and north pacific spiny dogfish (*Squalus suckleyi*, Family: *Squalidae*, Order: *Squaliformes*, GenBank: JN419081.1). Based on this sequence information, we designed PCR primers for amplification of IgNAR genes of cloudy catshark. Additionally, we designed PCR primers based on sequences with high homology to the VNAR gene sequences of nurse shark, banded houndshark, and small-spotted catshark. Using these primers, we performed PCR reactions with cDNA derived from cloudy catshark as a template and analyzed the gene sequences of the amplified PCR fragments. Finally, we obtained the cloudy catshark IgNAR gene sequence (DNA data bank of Japan (DDBJ): LC842168).

As a result of comparing the obtained amino acid sequence of IgNAR derived from the cloudy catshark (Figure S1) with the amino acid sequences of IgNAR from the other five sharks, it was revealed that the identity of the C4 region among the constant regions C1, C2, C3, C4, and C5 is the highest (Table 1, Figure S2). In addition, it was confirmed that the cysteine residues, which are commonly conserved in the constant regions of IgNAR from the five species of sharks, are also conserved in the IgNAR derived from the cloudy catshark (Figure S1).

### 2.2. Expression and Purification of the C4-Domain Recombinant Protein Derived from Cloudy Catshark

**Figure 1:**
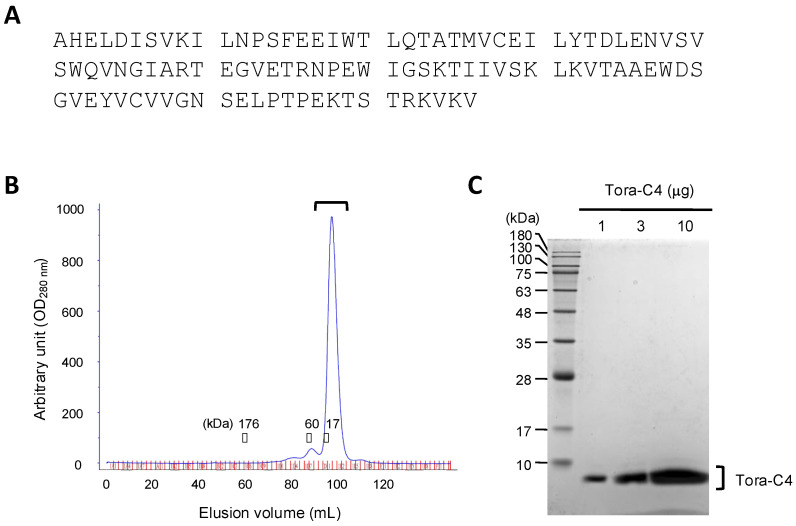
Expression and purification of the C4 domain (Tora-C4) recombinant protein of the cloudy catshark. (**A**) The amino acid sequence of the Tora-C4 recombinant protein expressed in *E. coli*. (**B**) Purification of the Tora-C4 recombinant protein on gel filtration. The Tora-C4 recombinant protein was expressed as inclusion bodies in *E. coli*, dissolved, and refolded, followed by purification on anion exchange column chromatography. The fraction containing the Tora-C4 recombinant protein was further purified on gel filtration. (**C**) Analysis of the purified Tora-C4 recombinant protein by SDS-PAGE. The purified Tora-C4 recombinant protein was resolved by SDS-PAGE, and the gel was stained with CBB.

If antibodies that can cross-recognize the IgNARs of not only the cloudy catshark but also other shark species could be obtained, it would enable IgNAR expression analysis across different shark species. Therefore, we designed and expressed the cloudy catshark C4-domain recombinant protein, which has the highest identity between the cloudy catshark and the other five shark species. Subsequently, we optimized the gene sequence encoding the C4 domain derived from the cloudy catshark (Tora-C4) (Figure 1A) to enhance its expression in *E. coli* and cloned the codon-optimized sequence into an *E. coli* protein expression vector, followed by a Maxi-Prep preparation of the plasmid. Next, we transformed *E. coli* Rosetta (DE3)/pLysS with this expression vector and induced the recombinant protein expression with IPTG. As a result, it was revealed that the recombinant protein of the Tora-C4 domain was produced as inclusion bodies within the *E. coli*.

Next, we washed the inclusion bodies with a buffer containing detergents for crude purification. Then, we solubilized the recombinant protein of the Tora-C4 domain using a buffer containing urea and guanidine, and performed rapid dilution into a buffer containing arginine, allowing it to refold over three days. The resulting solution was dialyzed against Tris-HCl buffer and the Tora-C4 recombinant protein was purified through anion exchange column chromatography and gel filtration (Figure 1B).

The purified recombinant protein of the Tora-C4 domain was subjected to SDS-PAGE and the expression level and purity were confirmed by Coomassie brilliant blue (CBB) staining (Figure 1C).

### 2.3. Establishment of Anti-Tora-C4 Domain Serum and Monoclonal Antibodies

To produce polyclonal and monoclonal antibodies against the Tora-C4 domain, we immunized rats with the purified recombinant Tora-C4 domain protein. The immunization protocol included an injection of an emulsion of the purified recombinant Tora-C4 domain protein and the TiterMax Gold adjuvant. After sufficient antibody titers were confirmed in the plasma, peripheral blood was drawn and serum was prepared. In addition, lymph node cells from the immunized rats were harvested and fused with myeloma cells to establish hybridomas.

The hybridoma cells were grown in HAT medium in 96-well plates and screened for the production of antibodies specific to the Tora-C4 domain using enzyme-linked immunosorbent assay (ELISA). Positive clones producing anti-Tora-C4 antibodies were selected and further cloned by limiting dilution to ensure monoclonality.

### 2.4. Reactivity of Anti-Tora-C4 Serum to Tora-C4 Domain Recombinant Protein Assessed by ELISA

**Figure 2:**
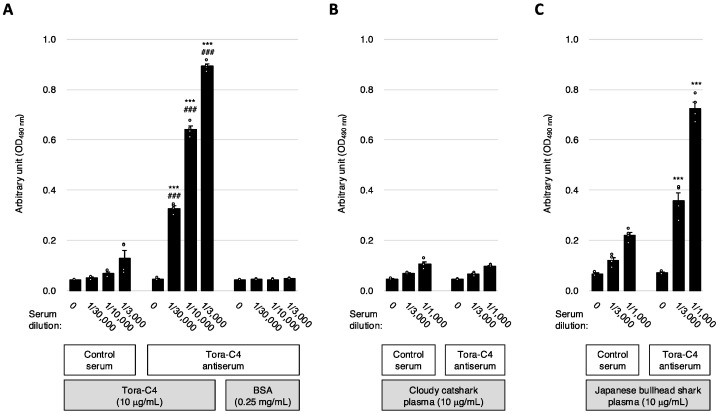
Analysis of IgNAR expression patterns by ELISA using anti-Tora-C4 serum. (**A**) The reactivity of anti-Tora-C4 serum against Tora-C4 recombinant protein and bovine serum albumin (BSA) was analyzed by ELISA. Data are shown as the mean ± S.E.M. (*n* = 4 samples). *** *p* < 0.001, Tukey–Kramer’s HSD test, Control serum vs. Tora-C4 antiserum in each serum dilution. ### *p* < 0.001, Tukey–Kramer’s HSD test, BSA vs. Tora-C4 in each Tora-C4 antiserum dilution. (**B**) The reactivity of anti-Tora-C4 serum against cloudy catshark plasma was analyzed by ELISA. Data are shown as the mean ± S.E.M. (*n* = 4 samples). (**C**) The reactivity of anti-Tora-C4 serum against Japanese bullhead shark plasma was analyzed by ELISA. Data are shown as the mean ± S.E.M. (*n* = 4 samples). *** *p* < 0.001, Student’s *t*-test, Control serum vs. Tora-C4 antiserum in each serum dilution.

First, the reactivity of the anti-Tora-C4 serum to the Tora-C4 domain recombinant protein was examined using an ELISA. The results showed that the anti-Tora-C4 serum reacted with the Tora-C4 protein in a concentration-dependent manner (Figure 2A). Next, the reactivity of the anti-Tora-C4 serum to plasma collected from non-immunized cloudy catshark was similarly assessed using ELISA. The results indicated no significant reactivity between the anti-Tora-C4 serum and the cloudy catshark plasma (Figure 2B).

Then, the reactivity of the anti-Tora-C4 serum to plasma collected from non-immunized Japanese bullhead shark was examined using ELISA. The results showed that the anti-Tora-C4 serum reacted with the plasma from the Japanese bullhead shark (Figure 2C), indicating that the anti-Tora-C4 serum reacts with the IgNAR present in the plasma of the Japanese bullhead shark. Thus, it was suggested that the anti-Tora-C4 serum could potentially detect IgNARs from sharks other than the cloudy catshark. Furthermore, it was indicated that the expression level of IgNAR in non-immunized cloudy catshark might be significantly lower compared to that in the Japanese bullhead shark.

### 2.5. Comparative Reactivity of Various Anti-Tora-C4 Monoclonal Antibodies to the Tora-C4 Domain Recombinant Protein by ELISA

**Figure 3:**
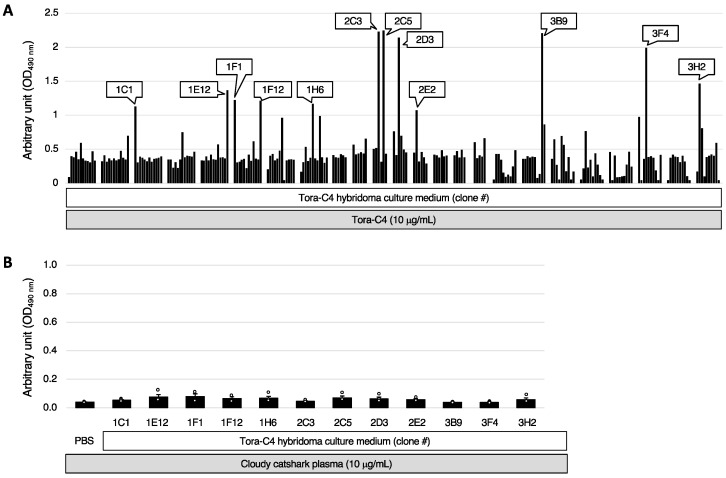
Analysis of IgNAR expression patterns by ELISA using anti-Tora-C4 mAb. (**A**) The reactivity of anti-Tora-C4 mAb against Tora-C4 recombinant protein was analyzed by ELISA. (**B**) The reactivity of 12 different anti-Tora-C4 mAbs against cloudy catshark plasma was analyzed by ELISA. Data are shown as the mean ± S.E.M. (*n* = 4 samples).

Next, the reactivity of various anti-Tora-C4 monoclonal antibodies (mAbs) to the Tora-C4 domain recombinant protein was compared using ELISA. The results showed that 12 clones (1C1, 1E12, 1F1, 1F12, 1H6, 2C3, 2C5, 2D3, 2E2, 3B9, 3F4, 3H2) exhibited high reactivity (Figure 3A). The culture supernatants of these 12 clones were then tested for reactivity with plasma derived from cloudy catshark using ELISA. Similar to the anti-Tora-C4 serum, none of the monoclonal antibodies showed reactivity to cloudy catshark plasma (Figure 3B).

### 2.6. Immunoblot Analysis with Selected Anti-Tora-C4 Monoclonal Antibodies

**Figure 4:**
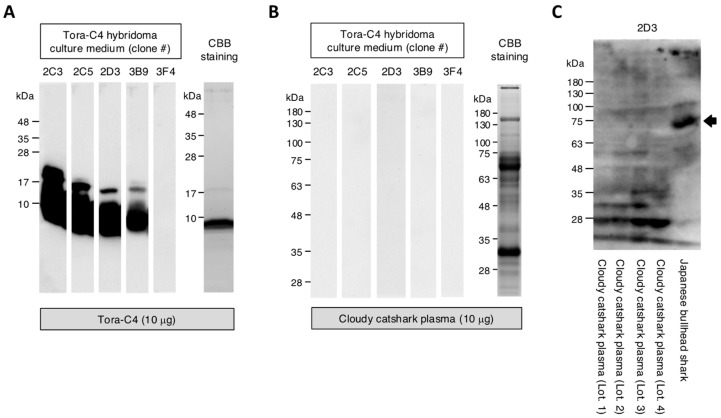
Analysis of IgNAR expression patterns by immunoblotting using anti-Tora-C4 mAb. (**A**) The reactivity of the culture supernatants (1/20 dilution) from five distinct Tora-C4 hybridoma clones against Tora-C4 recombinant protein was analyzed by immunoblotting. (**B**) The reactivity of the culture supernatants (1/20 dilution) from five Tora-C4 hybridoma clones against cloudy catshark plasma (10 μg) was determined by immunoblotting. (**C**) The reactivity of the culture supernatant (undiluted) from the 2D3 hybridoma clone against the plasma (30 μg) from different cloudy catshark individuals and Japanese bullhead shark plasma (30 μg) was determined by immunoblotting. The black arrow indicates a band of approximately 75 kDa, presumed to be Japanese bullhead shark-derived IgNAR.

Since monoclonal antibodies that could detect IgNAR in cloudy catshark plasma by ELISA were not obtained, further analyses were conducted using immunoblotting with five clones (2C3, 2C5, 2D3, 3B9, 3F4) that exhibited particularly high reactivity in ELISA. The results showed that four clones (2C3, 2C5, 2D3, 3B9) could detect the Tora-C4 protein (10 μg) at a dilution of 1/20 (Figure 4A). However, similar to the ELISA results, none of the clones reacted with cloudy catshark plasma (10 μg) even at a 1/20 dilution (Figure 4B).

### 2.7. Reactivity of 2D3 Clone to Plasma from Different Cloudy Catshark Individuals and Japanese Bullhead Shark

Further analysis was conducted using the culture supernatant of the 2D3 clone on plasma collected from four different individuals of cloudy catshark (30 μg each). The results showed that, while a band was observed around 30 kDa in the plasma from cloudy catshark, no band was detected around the expected molecular weight of IgNAR (~75 kDa). On the other hand, the analysis of plasma from Japanese bullhead shark (30 μg) with the 2D3 clone revealed a band around the expected molecular weight of IgNAR (approximately 75 kDa) (Figure 4C).

These findings suggest that non-immunized cloudy catshark may have a lower expression level of IgNAR compared to Japanese bullhead shark. The stability of IgNAR in cloudy catsharks may plausibly be lower, potentially leading to increased degradation, as indicated by the observation of a lower molecular weight band, however, this interpretation requires confirmation through future studies.

## 3. Discussion

In this study, we performed a sequence analysis of the IgNAR gene from the cloudy catshark, a small demersal shark, which was previously unexplored. We established both polyclonal and mAbs recognizing the constant region C4 domain, which shows the highest homology to known shark sequences, and conducted expression analysis of IgNAR. The anti-Tora-C4 serum did not react with the plasma from non-immunized cloudy catshark in ELISA but did react with the plasma from Japanese bullhead shark. Similarly, the anti-Tora-C4 mAbs did not react with cloudy catshark plasma in ELISA or immunoblotting. However, when using high concentrations of anti-Tora-C4 mAbs in immunoblotting, a band corresponding to the expected molecular weight of IgNAR (75 kDa) was detected in the plasma of Japanese bullhead shark. In contrast, a band around 30 kDa was detected in the plasma of cloudy catshark, which could be a degradation product containing the C4 domain.

These results raise two tentative possibilities. One is that the expression level of IgNAR in non-immunized cloudy catsharks might be lower than that observed in Japanese bullhead sharks. The other is that IgNAR in cloudy catsharks could exhibit reduced stability, potentially resulting in greater degradation after expression. While the established anti-Tora-C4 mAbs appear to have some capacity to react with IgNAR from bullhead sharks, additional studies are essential to confirm their cross-reactivity with IgNARs from other shark species. Future investigations will aim to detect IgNAR in plasma across diverse shark species and analyze IgNAR gene sequences, with the goal of expressing these in *E. coli* to verify the extent of homology in the C4 domain among species. Extensive further experimentation will be required to substantiate these preliminary hypotheses.

If high homology is confirmed, establishing mAbs with higher affinity to the C4 domain will be pursued. Alternatively, comparing the constant region sequences of IgNAR from multiple shark species may identify other regions with higher homology, leading to the development of antibodies targeting those epitopes. The current analysis utilized plasma from non-immunized cloudy catshark, where IgNAR expression was undetectable. Future work will examine changes in IgNAR expression in response to antigen sensitization in both cloudy catshark and Japanese bullhead shark. Evaluating whether antigen-specific IgNAR titers increase upon sensitization will also be crucial.

By determining conditions that efficiently induce IgNAR responses to antigen sensitization, we aim to identify suitable shark species and sensitization methods for developing demersal shark-derived VNAR-domain antibodies. This knowledge will significantly advance the development of demersal shark-derived VNAR-domain antibodies, potentially leading to major breakthroughs in this field.

## 4. Materials and Methods

### 4.1. Analysis of IgNAR Gene Sequence Derived from Cloudy Catshark

Cloudy catsharks and Japanese bullhead sharks inhabiting the coastal waters of Japan were captured and subsequently reared and bred in land-based sea water tanks. Peripheral blood was collected from the tail vein of cloudy catsharks and Japanese bullhead sharks without the need for euthanasia, and after heparin treatment (Heparin Sodium Injection 5000 units/5 mL, Mochida Pharmaceutical Co., Ltd., Shinjuku-ku, Tokyo, Japan), RNA was purified using Sepasol RNA I Super G (Cat. No.: 09379-84, Nacalai Tesque Inc., Nakagyo-ku, Kyoto, Japan). The purified RNA was then used as a template for cDNA synthesis using the PrimeScript™ 1st strand cDNA Synthesis Kit (Cat. No.: 6110A, Takara Bio Inc., Kusatsu, Shiga, Japan), and the resulting cDNA was subjected to gene sequence analysis.

### 4.2. Analysis of the Constant Region C2–C5 Domain Gene Sequences

From the RIKEN database (Squalomix: elasmobranch sequence archive by Laboratory for Phyloinformatics (Kuraku Lab), RIKEN BDR) of cloudy catshark [*Scyliorhinus torazame*] Storazame v1.0, Transcriptome, gene sequences showing high homology with IgNAR sequences from the nurse shark (*Ginglymostoma cirratum*, Family: *Ginglymostomatidae*, Order: *Orectolobiformes*, GenBank: U18701.1), spotted wobbegong (*Orectolobus maculatus*, Family: *Orectolobidae*, Order: *Orectolobiformes*, GenBank: DQ268538.1), banded houndshark (*Triakis scyllium*, Family: *Triakidae*, Order: *Carcharhiniformes*, GenBank: AB557743.1), small-spotted catshark (*Scyliorhinus canicular*, Family: *Scyliorhinidae*, Order: *Carcharhiniformes*, GenBank: JX556022.1), and north pacific spiny dogfish (*Squalus suckleyi*, Family: *Squalidae*, Order: *Squaliformes*, GenBank: JN419081.1) were searched. Based on the obtained sequence information, sense primer (tC2-F2: 5′-CGACACTTTCAACTGAGGTTGC-3′) and antisense primer (tC5-R1: 5′-CGGCAATAACTTGTAATATACTGC-3′) were designed and synthesized. PCR reactions were performed using these primer pairs with cloudy catshark cDNA as the template, and the gene sequence from the middle of the C1 domain of IgNAR to the stop codon was obtained by analyzing the amplified PCR fragments.

### 4.3. Analysis of the VNAR-C2 Domain Gene Sequences

Based on IgNAR VNAR gene sequences from nurse shark (GenBank: U18701.1), banded houndshark (GenBank: AB557743.1), and small-spotted catshark (GenBank: JX556022.1), which show high homology, a sense primer (STO184: 5′-AAGTCGTTTTCTCTGCAAATC-3′) was designed and synthesized. Based on the results of the C2-C5 domain gene sequence analysis, an antisense primer (STO160: 5′-CATCCACTGTCCAAACGATG-3′) was also designed and synthesized. Using these primers with cloudy catshark cDNA as the template, PCR reactions were performed to obtain PCR fragments. Subsequently, the obtained PCR fragments were used as templates for a second PCR reaction with sense primer STO184 modified to include an EcoRI restriction site (STO185: 5′-GCCGAATTCAAGTCGTTTTCTCTGCAAATC-3′, with the EcoRI site underlined) and antisense primer STO160 modified to include an XhoI restriction site (STO181: 5′-GCCCTCGAGCATCCACTGTCCAAACGATG-3′, with the XhoI site underlined). The amplified PCR fragments were digested with EcoRI (Cat. No.: 1040A, Takara Bio Inc., Kusatsu, Shiga, Japan) and XhoI (Cat. No.: 1094A, Takara Bio Inc.) and then cloned into the pEGFP-C1 vector (Cat. No.: 632470, Takara Bio Inc.). Sequence analysis of the resulting clones confirmed the gene sequence from the C2 domain to the region containing the CDR3 of VNAR.

### 4.4. Analysis of the Signal Peptide and VNAR Gene Sequences

From the RIKEN database (Squalomix: elasmobranch sequence archive by Laboratory for Phyloinformatics (Kuraku Lab), RIKEN BDR) of cloudy catshark gene sequences ([*Scyliorhinus torazame*] Storazame v1.0, Genome), gene sequences showing high homology with the IgNAR sequence from banded houndshark (GenBank: AB557743.1) were identified, and a sense primer (STO207: 5′-TGCCATTACTGCAAGACTGG-3′) was designed and synthesized. Based on the results from the above gene sequence analysis, antisense primers (STO190: 5′-GCTCACTGCAATGCTTTC-3′ and STO193: 5′-GTAGGTGGCACTGTCCTCAAC-3′) were designed. PCR reactions were performed with these primer pairs using cloudy catshark cDNA as the template, and the amplified PCR fragments were cloned using TA-cloning (Cat. No.: 6028, Mighty TA-cloning Kit, Takara Bio). Gene sequence analysis of the clones confirmed the VNAR gene sequence from the region containing complementarity determining region 1 (CDR1) to just before CDR3.

Next, to analyze the gene sequence of the signal peptide, sense primer (STO245: 5′-GTGTCTTTAAACTTTTCTGATTTTATTC-3′) and antisense primer (STO215: 5′-CGCTTGTTACTGTTGGTTGC-3′) were designed and synthesized. PCR reactions were conducted with these primers using cloudy catshark cDNA as the template, and the amplified PCR fragments were cloned using TA-cloning. Gene sequence analysis of the resulting clones confirmed the gene sequence of the signal peptide.

### 4.5. Construction of the Recombinant Protein Expression Vector for Cloudy Catshark Tora-C4

To construct an *E. coli* expression vector for the Tora-C4 recombinant protein, the following Tora-C4 gene was synthesized by Integrated DNA Technologies (Coralville, IA). The synthesized gene was inserted into the EcoRI/SalI sites of the pLM1 vector kindly provided by Dr. David N. Garboczi (National Institute of Allergy and Infectious Diseases, NIH, Bethesda, MD) to create the *E. coli* expression vector pLM1-Tora-C4. The gene sequence is as follows: 5′-GAATTC**AATTAAGGAGGATATTAAA**ATGGCTCACGAACTGGACATCTCTGTTAAAATCCTGAACCCGTCTTTCGAAGAAATCTGGACCCTGCAGACCGCTACCATGGTTTGCGAAATCCTGTACACCGACCTGGAAAACGTTTCTGTTTCTTGGCAGGTTAACGGTATCGCTCGTACCGAAGGTGTTGAAACCCGTAACCCGGAATGGATCGGTTCTAAAACCATCATCGTTTCTAAACTGAAAGTTACCGCTGCTGAATGGGACTCTGGTGTTGAATACGTTTGCGTTGTTGGTAACTCTGAACTGCCGACCCCGGAAAAAACCTCTACCCGTAAAGTTAAAGTTTAAGTCGAC-3′, in which the underlined (dotted line) section represents the EcoRI restriction site; the underlined (waved line) section represents the SalI restriction site; the underlined (single line) section represents the start codon; the underlined (double line) section represents the stop codon; the bold section indicates the ribosome entry site.

### 4.6. Induction of the Tora-C4 Recombinant Protein as Inclusion Bodies

The pLM1-Tora-C4 vector was transformed into *E. coli* Rosetta (DE3)/pLysS (Cat. No.: 70954, Merck & Co., Rahway, NJ, USA) and cultured in 4000 mL LB medium (Cat. No.: 20068-75, Nacalai Tesque Inc., Nakagyo-ku, Kyoto, Japan) at 37 °C. Induction of Tora-C4 recombinant protein expression as inclusion bodies (IBs) was achieved by adding 1 mM IPTG (Cat. No.: 096-05143, FUJIFILM Wako Pure Chemical Corp., Chuo-ku, Osaka, Japan) and incubating at 37 °C for 18 h. After harvesting the cells, they were resuspended in 180 mL of resuspension buffer [50 mM Tris-HCl (pH 8.0) (Cat. No.: 06938-15, Nacalai Tesque Inc.), 25% *w*/*v* sucrose (Cat. No.: 190-00013, FIJUFILM Wako Pure Chemical Corp.), 1 mM EDTA (pH 8.0) (Cat. No.: 06894-14, Nacalai Tesque Inc.), 0.1% sodium azide (Cat. No.: 26628-22-8, FIJUFILM Wako Pure Chemical Corp.), 10 mM DTT (Cat. No.: 14128-04, Nacalai Tesque Inc.)], with the addition of 3 mL of 50 mg/mL lysozyme (Cat. No.: 105281, Merck), and stirred at room temperature for 1 h. Subsequently, 375 mL of lysis buffer [50 mM Tris-HCl (pH 8.0), 1% *v*/*v* Triton-X100 (Cat. No.: 35501-02, Nacalai Tesque Inc.), 1% *w*/*v* sodium deoxycholate (Cat. No.: 10712-12, Nacalai Tesque Inc.), 100 mM NaCl, 0.1% sodium azide, 10 mM DTT, 1 mM EDTA (pH 8.0)] was added and stirred at room temperature for 1 h. Following this, 1.5 mL of DNase I (Cat. No.: 69182, Merck)) (2 mg/mL in 50% glycerol with 75 mM NaCl) and 6.3 mL of 1 M MgCl_2_ (Cat. No.: 20909-55, Nacalai Tesque Inc.) (final concentration: 6 mM) were added and stirred at room temperature for 1 h. The IBs were then recovered by centrifugation at 8670× *g* for 10 min at 4 °C. The IBs were washed with 50 mL of wash buffer [50 mM Tris-HCl (pH 8.0), 0.5% *v*/*v* Triton-X100, 100 mM NaCl, 0.1% sodium azide, 1 mM DTT, 1 mM EDTA (pH 8.0)] and resuspended using a homogenizer. After centrifugation at 8670× *g* for 10 min at 4 °C, the IBs were again resuspended and washed with an additional 50 mL of wash buffer. Next, the IBs were resuspended and washed with 30 mL of rinse buffer [50 mM Tris-HCl (pH 8.0), 0.1% sodium azide, 1 mM DTT, 1 mM EDTA (pH 8.0)]. Finally, the IBs were solubilized in 30 mL of guanidine buffer [25 mM MES buffer (pH 6.0) (Cat. No.: 21623-84, Nacalai Tesque Inc.), 10 mM EDTA (pH 8.0), 6 M guanidine HCl (Cat. No.: 17318-95, Nacalai Tesque Inc.), 1 mM DTT].

### 4.7. Refolding and Purification of Tora-C4 Recombinant Protein Inclusion Bodies

The IBs solubilized in 4 mL of guanidine buffer were further diluted with 50 mL of guanidine buffer and then added to 2 L of refolding buffer [1 M arginine (Cat. No.: 03321-65, Nacalai Tesque Inc.), 100 mM Tris-HCl (pH 8.0), 2 mM EDTA, 0.25 mM reduced glutathione (Cat. No.: 104090, Merck), 0.25 mM oxidized glutathione (Cat. No.: 27025-41-8, Merck), 0.5 M sucrose]. The mixture was stirred at 4 °C for 24 h. After refolding, the solution was transferred to a dialysis tube (molecular weight cut-off: 8 kDa, Cat. No.: 132584, Repligen, Boston, MA, USA) and dialyzed against 10 L of Milli-Q water for 12 h. Following this, the solution was dialyzed at 4 °C against 10 L of 10 mM Tris-HCl buffer (pH 8.0) for a total of 4 cycles, each lasting 12 h. Subsequently, 50 mL of DE52 anion exchange resin (Merck) in 10 mM Tris-HCl buffer (pH 9.0) was added and stirred at 4 °C for 1 h. The resin, which adsorbed the Tora-C4 recombinant protein, was packed into a Q Sepharose HP column (16/800) (Cat. No.: 17101403, Cytiva, Shinjuku-ku, Tokyo, Japan). Tora-C4 recombinant protein was eluted from the column by a gradient of 10 mM Tris-HCl buffer (pH 8.0) to 1 M NaCl in 10 mM Tris-HCl buffer (pH 8.0) (1 fraction = 8 mL, flow rate = 2 mL/min). Fractions containing Tora-C4 recombinant protein were further purified using gel filtration chromatography (D-PBS, pH 7.4, 1 fraction = 5 mL, flow rate = 2 mL/min, Superdex 200 pg, 26/60, Cat. No.: 28989336, Cytiva, Shinjuku-ku, Tokyo, Japan). The concentration of the purified Tora-C4 recombinant protein was measured by absorbance at 280 nm. The protein was then concentrated to 1 mg/mL using an Amicon Ultra centrifugal filter (Cat. No.: 36100101, molecular weight cut-off: 10 kDa, Merck).

### 4.8. Preparation of Rat Anti-Tora-C4 Serum and Tora-C4 Antibody-Producing Hybridomas

To prepare anti-Tora-C4 serum and Tora-C4 antibody-producing hybridomas, Tora-C4 recombinant protein (0.4 mg per rat) was mixed with TiterMax Gold (Titer Max, Norcross, GA, USA) to form an emulsion, which was then used to immunize Jcl:Wistar rats (7 weeks old, female, CLEA Japan, Inc., Meguro-ku, Tokyo, Japan). Three weeks later, whole blood was collected to obtain anti-Tora-C4 serum. Simultaneously, iliac lymph nodes were collected, and lymph node cells were fused with SP2/O myeloma cells to derivate hybridomas. The culture supernatants from the resulting hybridomas were used as anti-Tora-C4 monoclonal antibodies (mAbs) for IgNAR expression analysis.

### 4.9. ELISA (Enzyme-Linked Immunosorbent Assay)

Tora-C4 recombinant protein (10 µg/mL), cloudy catshark plasma (10 µg/mL), Japanese bullhead shark plasma (10 µg/mL), or BSA (0.25 mg/mL, Cat. No.: 01860-07, Nacalai Tesque Inc.) was added to a 96-well plate (Cat. No.: 353072, Corning, Corning, NY, USA) and incubated at room temperature for 2 h. After removing each solution, 5% skim milk (Cat. No.: 31149-75, Nacalai Tesque Inc.)/0.1% Tween 20 (Cat. No.: 23926-35, Nacalai Tesque Inc.)/Tris-buffered saline (TBS, Cat. No.: 35438-81, Nacalai Tesque Inc.) was added and blocked the plastic surface of the wells overnight at 4 °C. After washing with 0.1% Tween 20/TBS, Tora-C4 antiserum, control antiserum, or Tora-C4 mAb was added and incubated at room temperature for 2 h. Following washing with 0.1% Tween 20/TBS, peroxidase-labeled antibody to Rat IgG (H + L) (mouse serum-adsorbed) (SeraCare KPL Cat# 5220-0365, RRID: AB_3668946) was added and incubated at room temperature for 1 h. After washing with 0.1% Tween 20/TBS, the detection solution [10 mg o-phenylenediamine (Cat. No.: P8287-100TAB, Merck)/25 mL of 50 mM citrate-phosphate buffer (pH 5.0, Cat. No.: 31404-15, Nacalai Tesque Inc.), 5 µL H_2_O_2_ (Cat. No.: 20779-65, Nacalai Tesque Inc.)] was added and incubated at room temperature. The reaction was stopped with 2 N sulfuric acid (Cat. No.: 32520-55, Nacalai Tesque Inc.), and the absorbance was measured at 490 nm on a NIVO multiplate reader (Revvity, Waltham, MA, USA).

### 4.10. Immunoblot Analysis

After resolving 10 µg of Tora-C4 recombinant protein or 10 µg or 30 µg of cloudy catshark or Japanese bullhead shark plasma using SDS-PAGE, the proteins were transferred to a PVDF membrane (Cat. No.: 1214588, GVS Japan Co., Ltd., Shinjuku-ku, Tokyo, Japan), or the gel was directly stained with CBB Stain One (Cat. No.: 04543-51, Nacalai Tesque Inc.). The PVDF membrane was then treated with anti-Tora-C4 mAb, followed by reaction with peroxidase-labeled antibody to Rat IgG (H + L) (mouse serum-adsorbed), and detected using a ChemiDoc Touch (Bio-Rad, Hercules, CA, USA).

## Figures and Tables

**Table 1:** Conservation of the constant region amino acid sequences of IgNAR from six different shark species.

IgNAR Domain	C1	C2	C3	C4	C5
Identity (%)	43.9	40.6	47.2	56.3	52.8

The amino acid sequence identity of the IgNAR constant regions was analyzed using Snap Gene^®^ 5.2.4 for the following six species: cloudy catshark (*Scyliorhinus torazame*, Family: *Scyliorhinidae*, Order: *Carcharhiniformes*, DNA data bank of Japan (DDBJ): LC842168), nurse shark (*Ginglymostoma cirratum*, Family: *Ginglymostomatidae*, Order: *Orectolobiformes*, GenBank: U18701.1), spotted wobbegong (*Orectolobus maculatus*, Family: *Orectolobidae*, Order: *Orectolobiformes*, GenBank: DQ268538.1), banded houndshark (*Triakis scyllium*, Family: *Triakidae*, Order: *Carcharhiniformes*, GenBank: AB557743.1), small-spotted catshark (*Scyliorhinus canicular*, Family: *Scyliorhinidae*, Order: *Carcharhiniformes*, GenBank: JX556022.1), and North Pacific spiny dogfish (*Squalus suckleyi*, Family: *Squalidae*, Order: *Squaliformes*, GenBank: JN419081.1).

## Data Availability

The data presented in this study are available on request from the corresponding author.

## Supplementary Materials

The following supporting information can be downloaded at: https://www.mdpi.com/article/10.3390/md23010028/s1, Figure S1: Analysis of IgNAR derived from cloudy catshark. Figure S2: Sequence alignment of the constant region amino acid sequences of IgNAR from six different shark species.

## References

[1] Lu R.-M., Hwang Y.-C., Liu I.-J., Lee C.-C., Tsai H.-Z., Li H.-J., Wu H.-C. (2020). Development of Therapeutic Antibodies for the Treatment of Diseases. J. Biomed. Sci..

[2] Brekke O.H., Sandlie I. (2003). Therapeutic Antibodies for Human Diseases at the Dawn of the Twenty-First Century. Nat. Rev. Drug Discov..

[3] Beck A., Wurch T., Bailly C., Corvaia N. (2010). Strategies and Challenges for the next Generation of Therapeutic Antibodies. Nat. Rev. Immunol..

[4] Weisser N.E., Hall J.C. (2009). Applications of Single-Chain Variable Fragment Antibodies in Therapeutics and Diagnostics. Biotechnol. Adv..

[5] Holliger P., Hudson P.J. (2005). Engineered Antibody Fragments and the Rise of Single Domains. Nat. Biotechnol..

[6] Juma S.N., Gong X., Hu S., Lv Z., Shao J., Liu L., Chen G. (2021). Shark New Antigen Receptor (IgNAR): Structure, Characteristics and Potential Biomedical Applications. Cells.

[7] Pillay T.S., Muyldermans S. (2021). Application of Single-Domain Antibodies (“Nanobodies”) to Laboratory Diagnosis. Ann. Lab. Med..

[8] Wernery U. (2001). Camelid Immunoglobulins and Their Importance for the New-Born—A Review. J. Vet. Med. Ser. B.

[9] Arbabi-Ghahroudi M. (2017). Camelid Single-Domain Antibodies: Historical Perspective and Future Outlook. Front. Immunol..

[10] Hamers-Casterman C., Atarhouch T., Muyldermans S., Robinson G., Hammers C., Songa E.B., Bendahman N., Hammers R. (1993). Naturally Occurring Antibodies Devoid of Light Chains. Nature.

[11] Jovčevska I., Muyldermans S. (2020). The Therapeutic Potential of Nanobodies. BioDrugs.

[12] Jin B., Odongo S., Radwanska M., Magez S. (2023). NANOBODIES^®^: A Review of Diagnostic and Therapeutic Applications. Int. J. Mol. Sci..

[13] Steeland S., Vandenbroucke R.E., Libert C. (2016). Nanobodies as Therapeutics: Big Opportunities for Small Antibodies. Drug Discov. Today.

[14] Ingram J.R., Schmidt F.I., Ploegh H.L. (2018). Exploiting Nanobodies’ Singular Traits. Annu. Rev. Immunol..

[15] Muyldermans S. (2013). Nanobodies: Natural Single-Domain Antibodies. Annu. Rev. Biochem..

[16] Feige M.J., Gräwert M.A., Marcinowski M., Hennig J., Behnke J., Ausländer D., Herold E.M., Peschek J., Castro C.D., Flajnik M. (2014). The Structural Analysis of Shark IgNAR Antibodies Reveals Evolutionary Principles of Immunoglobulins. Proc. Natl. Acad. Sci. USA.

[17] Streltsov V.A., Varghese J.N., Carmichael J.A., Irving R.A., Hudson P.J., Nuttall S.D. (2004). Structural Evidence for Evolution of Shark Ig New Antigen Receptor Variable Domain Antibodies from a Cell-Surface Receptor. Proc. Natl. Acad. Sci. USA.

[18] Greenberg A.S., Avila D., Hughes M., Hughes A., McKinney E.C., Flajnik M.F. (1995). A New Antigen Receptor Gene Family That Undergoes Rearrangement and Extensive Somatic Diversification in Sharks. Nature.

[19] Hsu E. (2016). Assembly and Expression of Shark Ig Genes. J. Immunol..

[20] Dooley H. (2003). Selection and Characterization of Naturally Occurring Single-Domain (IgNAR) Antibody Fragments from Immunized Sharks by Phage Display. Mol. Immunol..

[21] Dooley H., Flajnik M.F. (2006). Antibody Repertoire Development in Cartilaginous Fish. Dev. Comp. Immunol..

[22] Matz H., Munir D., Logue J., Dooley H. (2021). The Immunoglobulins of Cartilaginous Fishes. Dev. Comp. Immunol..

[23] Takeda H., Ozawa T., Zenke H., Ohnuki Y., Umeda Y., Zhou W., Tomoda H., Takechi A., Narita K., Shimizu T. (2023). VNAR Development through Antigen Immunization of Japanese Topeshark (*Hemitriakis japanica*). Front. Bioeng. Biotechnol..

[24] Chen W.-H., Hajduczki A., Martinez E.J., Bai H., Matz H., Hill T.M., Lewitus E., Chang W.C., Dawit L., Peterson C.E. (2023). Shark Nanobodies with Potent SARS-CoV-2 Neutralizing Activity and Broad Sarbecovirus Reactivity. Nat. Commun..

[25] Matz H., Dooley H. (2019). Shark IgNAR-Derived Binding Domains as Potential Diagnostic and Therapeutic Agents. Dev. Comp. Immunol..

[26] Buffington J., Duan Z., Kwon H.J., Hong J., Li D., Feng M., Xie H., Ho M. (2023). Identification of Nurse Shark VNAR Single-domain Antibodies Targeting the Spike S2 Subunit of SARS-CoV-2. FASEB J..

[27] Jiang X., Sun S., Li Z., Chen M. (2023). Isolation and Characterization of Targeting-HBsAg VNAR Single Domain Antibodies from Whitespotted Bamboo Sharks (*Chiloscyllium plagiosum*). Mar. Drugs.

[28] Naylor G., Caira J., Jensen K., Rosana K., Straube N., Lakner C., Carrier J., Musick J., Heithaus M. (2012). Elasmobranch Phylogeny: A Mitochondrial Estimate Based on 595 Species. Biology of Sharks and Their Relatives.

[29] Jia L., Wang Y., Shen Y., Zhong B., Luo Z., Yang J., Chen G., Jiang X., Chen J., Lyu Z. (2023). IgNAR Characterization and Gene Loci Identification in Whitespotted Bamboo Shark (*Chiloscyllium plagiosum*) Genome. Fish. Shellfish Immunol..

[30] Lee S.S., Fitch D., Flajnik M.F., Hsu E. (2000). Rearrangement of Immunoglobulin Genes in Shark Germ Cells. J. Exp. Med..

[31] Kokubu F., Litman R., Shamblott M.J., Hinds K., Litman G.W. (1988). Diverse Organization of Immunoglobulin VH Gene Loci in a Primitive Vertebrate. EMBO J..

