# Supplementary material for: Expression Analysis of Heavy-Chain-Only Antibodies in Cloudy Catshark and Japanese Bullhead Shark
Source: Mar Drugs. 2025 Jan 8;23(1):28. doi: 10.3390/md23010028 (PMC11767079; doi:10.3390/md23010028)
Supplement: Supplementary file 1 [file marinedrugs-23-00028-s001.zip › marinedrugs-3293428-supplementary.pdf]

```
              ↱ VNAR
MKLYIIIFPRSGAFTARVEQTPRYATKYIGESLTINCAYIDSSYPLTSTYWYRTELGSTDKESISIG
--------------
 Signal sequence
                                                     ↱ C1
GRYDVSVNKRAKTFSLRIGDLRVEDSATYICGTPLGLGFSDGYGSGTMLTVNARPTAPTSAPTISLQ

              *
ISEELKATGFVQLLCLISGYYPESIAVSWEKSEKIIESGVTTTLPAKSSGGLYSSTSILKLPLQEWG

      *                   ↱ C2                   *
SGSVYSCQVTHSATNSNKRKEIRSISELAVFLRDPSVEGMWRNKTATLLCEVVSTLSTEVAIVWTVD

                                        *                   ↱ C3
GRRMTDGVRTEASTKEGNQYLTISRLTSSVKEWDSGAEYNCSAQQGQSSTPISKRTGQIKADPKKPN

                    *
LRLLPPSPEDIQSSSSATLTCLVRGFYPDRIAISWEKNGVDLTSKSTTFHSALEQDHTFSTSSLLIL

            *                      ↱ C4                      *
PSIEWKSGAKYTCTAFHLPSQSNETRSISYPKGDCHELDISVKILNPSFEEIWTLQTATMVCEILYT

                                                    *
DLENVSVSWQVNGIARTEGVETRNPEWIGSKTIIVSKLKVTAAEWDSGVEYVCVVGNSELPTPEKTS

      ↱ C5                        *
TRKVKVGEMHPPKVYILPPSMDEINTEKTATLVCLAIGFYPTEIYVAWMVNDTLLDADYPCQLECGK

                       *
GNGSFSSRLRVTAAEWDSGNNYSCLVGHPSLKRNLIRSINKSQGKPTLVNVSLVLTDSFKSCI
```

Figure S1. Analysis of IgNAR derived from cloudy catshark. The amino acid sequence of IgNAR from cloudy catshark. The underline (dotted line) indicates the signal sequence. Upwards arrows with tip rightwards indicate the start positions of the variable region VNAR and the constant regions C1, C2, C3, C4, and C5. The asterisk (*) denotes conserved cysteine residues within each domain of the constant regions.

C1

```
                              *                                                          *
cloudy catshark              TAPTSAPTISLQIS--EELKATGFVQLLCLISGYYPESIAVSWEKSEKIIESGVTTTLPAKSSGGLYSSTSILKLPLQEWGSGSVYSCQVTHSATNSNKRKEIRSIS
North Pacific spiny dogfish  APQPTPPIISLLYSATDELREKGFVQLVCLISEYQPESIGVSWEKNGNAIQSGFTTSSAAKNSNGDFSSTSLLQVPLQEWASGSVYTCQVSHSPTSSNQRKEIRSTS
spotted wobbegong            GVQPSPPVISLLYSATEEQRGNGFVQLICLISGYYPENIAVSWQKNRNDISSGFTTSPSMKTSTNDFSSTSLLNVPLQEWSSGSVYSCRVSHSATNSNQRKEIRSTS
nurse shark                  GIPPSPPIVSLLHSATEEQRANRFVQLVCLISGYYPENIAVSWQKNTKTITSGFATTSPVKTSSNDFSCASLLKVPLQEWSRGSVYSCQVSHSATSSNQRKEIRSTS
banded houndshark            G--PTPPIINL-FSETDELRAKGFVQLICLISEYKPESIRVSWEKNGNARQSGFTTTSPCKTAKGEFQSRSILTLPLQEWNSGSTYSCQVTHSATNSNKRKEIRSTS
small-spotted catshark       G--RAAPTISLLIS--EEMKATGFVQLLCLISGYYPERIAVSWEKSGNIVESGVTTTPLTKSSDGLYSSTSFLKLPLQEWGSDSAYSCQVTHSATNSNTRKEIRSTS
```

C2

```
cloudy catshark              ELAVFLRDPSVEGMWRNKTATLLCEVVSTLSTEVAIVWTVDGRRMTDGVRTEASTKEGNQYLTISRLTSSVKEWDSGAEYNCSAQQGQSSTPISKRTGQIK
North Pacific spiny dogfish  ELAVFLRDPSVEEIWINKTATLVCEVVSTVPTEVAISWTVDGKMRTKGVLTEPATKYGDQYLTIGRLTSSVEEWESGIEYSCSAQEGQSSTAVSQRTGKAK
spotted wobbegong            EIAVFLRDPSVEEIWINKSATVVCEVLSTVSTGVVISWMVDGKVRTEGVRIEAAKMDGNQYLTISRLSSSVEEWQSGVEYTCSAKQDQSSTPVSKRTGKTK
nurse shark                  EIAVLLRDPTVEEIWIDKSATLVCEVLSTVSAGVVVSWMVNGKVRNEGVQMEPTKMSGNQYLTISRLTSSVEEWQSGVEYTCSAKQDQSSTPVVKRTRKAR
banded houndshark            EITVFLRDPSLEEIWIKKTVTLICEVVSTVPSVVGISWTVDGKKRTEGVQIEGRQQGQNQYLTISRLTSSVEEWDRGAEYNCSAQQSESSTPVSKHTQKLK
small-spotted catshark       ELALFLRDPSVEGMWLNKTATLMCEVVSTLSTEVAIVWIVNGQTRTEGVRTEATTTDGDQYLTISRLTSTVEEWDSGAEYKCSAQQGQSATPISKSTGQIK
```

C3

```
cloudy catshark              ADPKKPNLRLLPPSPEDIQSSSSATLTCLVRGFYPDRIAISWEKNGVDLTSKSTTFHSALEQDHTFSTSSLLILPSIEWKSGAKYTCTAFHLPSQSNETRSISYPK
North Pacific spiny dogfish  VEPVKPKLRLLPPSPEEIQSTSAATLTCLIRGFYPDNIIVSWEKDGAALSANVTSFPTALEQDLTFSTRSLLTLPSAEWKRGSTYTCAASHPPSQSTVKGSISSPK
spotted wobbegong            VEPMKPYLRLLPPSPEEIQNISSAILTCLIRGFYPDKIRVSWEKDGAAVSGNITSFPTALEQDLTFSTRSLLILPAVEWKSGAKYTCIASHPPSQSTVKRVIRSPK
nurse shark                  VEPTKPHLRLLPPSPEEIQSTSSATLTCLIRGFYPDKVSVSWQKDDVSVSANVTNFPTALEQDLTFSTRSLLNLTAVEWKSGAKYTCTASHPPSQSTVKRVIRNQK
banded houndshark            VKPSKPNLRLLPPSAEELQSSSVATLTCLIRGFYPDKISISWEKDGAVLSSNITRFPTALEQDQTFSTSSLLILPAGEWKTGARYTCTASHPASKFTGKRTINSPK
small-spotted catshark       AEPTKPILRLLPPSPEEIRSSNSATLTCLVRGFYPDIITISWEKNGVDLTSKSTTFHSSLEQHQTFSTSSLLILPSMEWKTGAKYTCTAFHLPSQSNETRSISYPK
```

C4

```
cloudy catshark              HELDISVKILNPSFEEIWTLQTATMVCEILYTDLENVSVSWQVNGIARTEGVETRNPEWIGSKTIIVSKLKVTAAEWDSGVEYVCVVGNSELPTPEKTSTRK
North Pacific spiny dogfish  HEADISVKILNPPFEEIWTQRTATIVCEVVYSDLENVSVSWQVDGSRRTEGVETRTPEWSGSKSAIVSKLKVTRAEWESGVEYLCFVEDSALPTPVKISTRK
spotted wobbegong            GQPDISVNLLNPPFEEIWTQKTATIVCEIVYSDLENVNVFWQVNGSERTEGVETQNPEWSGSKSTIVSKLKVTSSEWDSGVEYVCLVEDSELPTPVKSSIRK
nurse shark                  RQTDISVSLLKPPFEEIWTQQTATIVCEIVYSDLENIKVFWQVNGVERKKGVETQNPEWSGSKSTIVSKLKVMASEWDSGTEYVCLVEDSELPTPVKASIRK
banded houndshark            YEEDISVNILNPSFEEIWVQKTATIVCEIRYTVLENVSVSWQVDGRMRTEGVETQTPEWSGSKTTIMSKLKVTAAEWDTGVEYVCLAEGSELPTPKKRSTRK
small-spotted catshark       HELDISVKILNPSFEEIWTQQTATMVCEILYTDLENVSVSWQVNGTARTEGVETRNPEWIGSKSIIVSKLKVTAAEWDSGVEYVCVVGNSELPTPEKTSTRK
```

C5

```
cloudy catshark              GEMHPPKVYILPPSMDEINTEKTATLVCLAIGFYPTEIYVAWMVNDTLLDADYPCQLECGKGNGS--FSSRLRVTAAEWDSGNNYSCLVGHPSLKRNLIRSINKSQ
North Pacific spiny dogfish  GEMYPPKVYVLPPSADEIDTENTATLVCLATGFYPAEIYIAWMANDTLLDSAYPSQPDTEKTNGSNSIGSRLRLTAAEWNSGTTYSCLVGHPSLKMNLIRSINKSH
spotted wobbegong            REMYPPKVYVLHPSTDEIDTENSATLVCLATGFSPAEIYVGWMANDTLLNSGYRSQVENEKGNGSNFIINRLRLTAAEWDSDTTYSCLVGHPSLSRDLIRSINKSH
nurse shark                  SQMHPPKVYLLHPSTDEIDTENSATLMCLATNFHPAEIYVGWMANDTLLDSGYRTQVDSEKGSGSSFVTDRLRLTAAEWNSDTTYSCLVGHPSLNRDLIRSTNKSN
banded houndshark            GAMNSPKVYILPPSVAEIDSEKTATLMCLATGFYPAEIYIAWLANDTLLDSDFPNQPVSEKGNGSSFIASRLRLTAAEWNTGTTYSCLVGHPSLERNLIRSINKSY
small-spotted catshark       GEMHPPKVYVLPPSIDEIDTEKTATLVCLATDFYPTEIYVAWMVNDTLLDADYPFELECGKGNGS--FSSRLRVTAAEWNSGNNYSCLVGHPSLKRNLIRSINKSF
```

Figure S2. Sequence alignment of the constant region amino acid sequences of IgNAR from six different shark species. The amino acid sequence identity of the IgNAR constant regions was analyzed using Snap Gene® 5.2.4 for the following six species: cloudy catshark, nurse shark, spotted wobbegong, banded houndshark, small-spotted catshark, and North Pacific spiny dogfish. Conserved amino acids among six species are highlighted as yellow. The asterisk (*) denotes conserved cysteine residues within the constant region.
